# Can bioelectrical impedance analysis effectively reflect the nutritional status of intensive care unit patients?

**DOI:** 10.1186/2197-425X-3-S1-A581

**Published:** 2015-10-01

**Authors:** A Taggu, B Krishna, S Sampath, SR Wudaru

**Affiliations:** St. Johns Medical College Hospital, Critical Care Medicine, Bangalore, India

## Introduction

Use of Bioelectrical Impedance Analysis (BIA) to assess nutritional status in intensive care unit (ICU) patients has not been extensively studied.Traditionally used clinical and biochemical indicators are not reliable in critically ill patients [1].

## Objectives

To test the hypothesis that BIA indices can reliably evaluate nutritional status in ICU patients.

## Methods

A prospective observational study done in patients admitted to medical ICU between 1st October and 31st December 2014. After informed consent, all patients > 18 years were included. Exclusion criteria were pregnancy, amputees, Body mass Index > 34 and < 16 Kg/m^2^, those with cardiac pacemakers and ascites [2]. Baseline demographics, clinical, biochemical and BIA data were recorded. Based on serum albumin (SA) and total lymphocyte count (TLC) [3], three nutritional status groups were formed. Well-nourished group (WNG) had SA ≥ 3.5 g/dl and TLC ≥ 1400 cells/m^3^ and severely malnourished group (SMNG) had SA < 2.8 g/dl and TLC < 1000 cells/mm^3^. Moderately malnourished group (MMNG) had values in between the two groups.

## Results

Out of the ninety patients, 66.7% (60/90) were in MMNG and 11% in SMNG. On comparison of traditional indicators with BIA, only SA, TLC and Haemoglobin were significant. BIA indices like phase angle (PA) and Extracellular water (ECW)/ Total body water (TBW) had higher values in WNG and SMNG respectively. PA positively correlated with SA and ECW/TBW was negatively associated with SA and mechanical ventilation days.PA among non-survivors were significantly lower than survivors.

## Conclusions

BIA is a reliable tool for nutritional assessment in ICU patients. ECW/TBW and PA are important indices and can be used to prognosticate critically ill patients.Further studies in a larger number of patients are needed to validate this technique.

## Grant Acknowledgment

I am thankful to all the patients and the staff in Medical ICU for their support and cooperation.
